# Mycolactone Gene Expression Is Controlled by Strong SigA-Like Promoters with Utility in Studies of *Mycobacterium ulcerans* and Buruli Ulcer

**DOI:** 10.1371/journal.pntd.0000553

**Published:** 2009-11-24

**Authors:** Nicholas J. Tobias, Torsten Seemann, Sacha J. Pidot, Jessica L. Porter, Laurent Marsollier, Estelle Marion, Franck Letournel, Tasnim Zakir, Joseph Azuolas, John R. Wallace, Hui Hong, John K. Davies, Benjamin P. Howden, Paul D. R. Johnson, Grant A. Jenkin, Timothy P. Stinear

**Affiliations:** 1 Department of Microbiology, Monash University, Clayton, Victoria, Australia; 2 Victorian Bioinformatics Consortium, Monash University, Clayton, Victoria, Australia; 3 Department of Microbiology and Immunology, University of Melbourne, Parkville, Victoria, Australia; 4 Groupe d'Etude des Interactions Hôte-Pathogène, UPRES-EA 3142, Université d'Angers, Angers, France; 5 Laboratoire de Parasitologie-Mycologie, Centre Hospitalier Universitaire, Angers, France; 6 Laboratoire de Neurobiologie et Transgénèse, UPRES-EA 3143, Université d'Angers, Angers, France; 7 Department of Primary Industries, Mickleham Road, Attwood, Victoria, Australia; 8 Department of Biology, Millersville University, Millersville, Pennsylvania, United States of America; 9 Department of Biochemistry, University of Cambridge, Cambridge, United Kingdom; 10 Department of Infectious Diseases, Austin Health, Heidelberg, Victoria, Australia; Institut Pasteur, France

## Abstract

Mycolactone A/B is a lipophilic macrocyclic polyketide that is the primary virulence factor produced by Mycobacterium ulcerans, a human pathogen and the causative agent of Buruli ulcer. In M. ulcerans strain Agy99 the mycolactone polyketide synthase (PKS) locus spans a 120 kb region of a 174 kb megaplasmid. Here we have identified promoter regions of this PKS locus using GFP reporter assays, in silico analysis, primer extension, and site-directed mutagenesis. Transcription of the large PKS genes mlsA1 (51 kb), mlsA2 (7 kb) and mlsB (42 kb) is driven by a novel and powerful SigA-like promoter sequence situated 533 bp upstream of both the mlsA1 and mlsB initiation codons, which is also functional in Escherichia coli, Mycobacterium smegmatis and Mycobacterium marinum. Promoter regions were also identified upstream of the putative mycolactone accessory genes mup045 and mup053. We transformed M. ulcerans with a GFP-reporter plasmid under the control of the mls promoter to produce a highly green-fluorescent bacterium. The strain remained virulent, producing both GFP and mycolactone and causing ulcerative disease in mice. Mosquitoes have been proposed as a potential vector of M. ulcerans so we utilized M. ulcerans-GFP in microcosm feeding experiments with captured mosquito larvae. M. ulcerans-GFP accumulated within the mouth and midgut of the insect over four instars, whereas the closely related, non-mycolactone-producing species M. marinum harbouring the same GFP reporter system did not. This is the first report to identify M. ulcerans toxin gene promoters, and we have used our findings to develop M. ulcerans-GFP, a strain in which fluorescence and toxin gene expression are linked, thus providing a tool for studying Buruli ulcer pathogenesis and potential transmission to humans.

## Author Summary

Buruli ulcer (BU) is a serious skin infection of humans predominantly occurring in West and Central Africa. The disease is caused by infection with *Mycobacterium ulcerans*, a bacterium that produces an unusual toxin called mycolactone. There are many unanswered questions surrounding BU, particularly regarding the role of mycolactone in disease and how *M. ulcerans* is transmitted to humans. Here, we have partly addressed these questions by identifying genetic factors controlling the transcription of the mycolactone genes. Using a variety of experimental approaches, including green fluorescent protein (GFP) as a reporter of gene expression, we have identified strong promoters that drive transcription of the mycolactone genes in *M. ulcerans*. We then used our GFP reporters to produce highly fluorescent *M. ulcerans-*GFP that were readily visualized by microscopy. Mosquitoes have been proposed as a potential vector of *M. ulcerans* so we used *M. ulcerans*-GFP in feeding experiments with mosquito larvae. *M. ulcerans*-GFP accumulated within the insects, whereas other mycobacteria did not. This is the first report of the mycolactone gene promoters, and we have used our findings to develop *M. ulcerans*-GFP, a strain in which fluorescence and toxin gene expression are linked, thus providing a powerful tool for studying Buruli ulcer.

## Introduction

*Mycobacterium ulcerans* is the causative agent of Buruli ulcer (BU) an emerging but neglected disease found predominantly in tropical regions of the world and with an increasing incidence in West and Central Africa [1],[2]. BU is a chronic infection of subcutaneous tissue that can result in high morbidity such as permanent scarring and functional disabilities. The combination of rifampin and an aminoglycoside for four to eight weeks leads to the healing of early lesions without radical surgery and is now the recommended standard regimen [3]. However, substantial tissue damage often necessitates surgery [4]. The social and economic burden of BU can be severe, particularly in impoverished rural regions of West Africa where the prevalence of BU is sometimes higher than that of the two most significant mycobacterial diseases, leprosy and tuberculosis. Cases of BU are usually clustered around swamps and slow-flowing water and while the mode of transmission of *M. ulcerans* is unknown, evidence to date suggests, fish [5], snails [6] and certain carnivorous aquatic insects [7],[8] can all harbour the bacterium. Recent studies in Australia suggest mosquitoes may play a role in transmission [9],[10].

A major factor influencing the pathology of Buruli ulcer is the production by *M. ulcerans* of a secondary metabolite called mycolactone [11]. Mycolactone is an immunosuppressive and cytotoxic macrocyclic polyketide, characterised by a 12-membered macrolactone core appended to a highly unsaturated acyl side chain [11],[12]. Polyketides are a class of naturally occurring compounds, some of which have potent pharmaceutical activity such as the immune suppressor rapamycin, the antibiotic erythromycin A, and the antiparasitic agent avermectin [13]–[15]. Why *M. ulcerans* produces mycolactone is unknown. However, studies on the effect of the molecule in cell culture and animal models have shown that in the microgram range it has cytotoxic properties, while at sub-cytotoxic concentrations it has immunomodulatory properties, most strikingly the inhibition of tumour necrosis factor production by monocytes and macrophages [16]–[18]. In mice, mycolactone has been shown to concentrate within peripheral blood monocytes [19].

**Figure 1:**
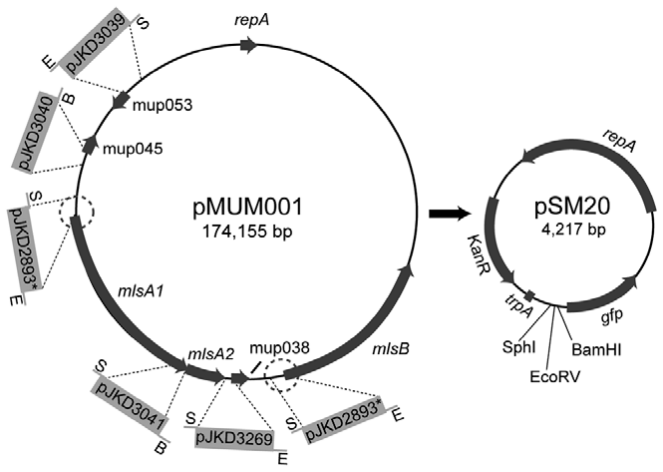
Schematic representation of the *M. ulcerans* Agy99 megaplasmid pMUM001, and the promoterless GFP vector pSM20. The regions upstream of key genes involved in mycolactone biosynthesis cloned into pSM20 and the names of the resulting plasmid GFP-reporter constructs are indicated. The 8.4 kb duplicated region that spans the load modules of *mlsA1* and *mlsB* and 1.6 kb upstream of each gene is circled. E = *Eco*RV, B = *Bam*HI, S = *Sph*I, ‘*’ indicates that this single construct represents the encircled duplicated regions upstream of *mlsA1* and *mlsB*.

Mycolactone synthesis is dependent on the pMUM megaplasmid found in *M. ulcerans* and closely related mycobacteria (Figure 1) [20]–[23]. This plasmid contains three, very large genes (*mlsA1*: 51 kb, *mlsA2*: 7 kb, and *mlsB*: 42 kb) that encode type I polyketide synthases (PKS). MlsA1 and MlsA2 synthesize the upper side chain and macrolactone core, whilst MlsB synthesizes the acyl side chain [22]. A putative beta-ketoacyl transferase encoded by another pMUM gene, mup045, is thought to catalyse the ester linkage between the acyl side chain and the macrolactone core whilst a P450 hydroxylase, encoded by mup053, oxidizes the side chain at C12′ (Figure 1) [22]–[25]. A third gene, mup038, is predicted to encode a type II thioesterase that might be required for removing aberrant polyketide extension products from the Mls PKS that form during synthesis. An unusual feature of the mycolactone PKS is the very high level of sequence identity between domains of the same function (98.7–100% nt identity and 98.3–100% aa identity). This observation suggested that the evolution of the locus may be recent and also prone to rearrangements that result in either loss of mycolactone production or production of new mycolactones. These hypotheses have recently gained support by studies that have shown (i) all mycolactone producing mycobacteria (which includes *M. ulcerans* and some closely related fish and frog pathogens) have recently evolved from a common *Mycobacterium marinum* ancestor by pMUM plasmid acquisition [23]–[28], (ii) laboratory passaging leads to mycolactone negative mutants through spontaneous deletion of *mls* gene fragments [29], and (iii) natural swapping of particular acyltransferase and ketoreductase domains and loss or gain of entire extension modules in some strains of *M. ulcerans* has led to the production of new mycolactones [30],[31].

However, there have been very few studies of gene expression in *M. ulcerans.* Therefore, in this study we began by investigation of the mycolactone-associated genes *mlsA1/mlsA2*, *mlsB*, mup045, mup053 and mup038. Promoter regions were mapped upstream of the above genes using a GFP reporter. Putative transcriptional start sites and promoter sequences were then identified by primer extension analysis and site-directed mutagenesis. The GFP reporter containing the promoter region of the *mls* genes was then used to transform *M. ulcerans*. This recombinant GFP *M. ulcerans* fluoresced brightly and was used to follow infection in both mice and mosquito larvae.

## Materials and Methods

### Bacterial strains and culture conditions

The bacterial strains and plasmids used in this study are described in Table S1. All cloning experiments were performed in *Escherichia coli* DH10B, cultivated in Luria-Bertani (LB) broth at 37°C or on LB agar containing 50 µg kanamycin, for 16 hours at 37°C. Mycobacterial strains were grown in Middlebrook 7H9 medium (Difco) supplemented with albumin (6.25% (w/v)), dextrose (2.5% (w/v)), sodium chloride (1.1% (w/v)), catalase (5×10^−4^% (w/v)) and 0.05% (v/v) Tween−80 at 37°C for *Mycobacterium smegmatis* and 30°C for *M. marinum* and *M. ulcerans*. Mycobacteria were also cultured on 7H10 agar supplemented with OADC (Difco). Recombinant mycobacteria were cultivated with kanamycin at a final concentration of 25 µg ml^−1^.

### DNA methods

Standard methods were used for cloning, PCR and DNA sequencing. The oligonucleotides used in this study for PCR, RT-PCR and DNA sequencing are listed in Table S2. Genomic DNA was extracted from mycobacteria as described [23]. The broad host range, promoterless, GFP (*gfpmut3*) vector pSM20, that replicates in *E. coli*, *Corynebacterium* sp. and *Mycobacterium* sp, was used for all promoter cloning experiments [32]. PCR products derived from the upstream regions were modified using oligonucleotides described in Table S2 and ligated into the unique restriction enzyme sites immediately upstream of the *gfp* gene in pSM20 (Figure 1). Constructs were confirmed to be correct by DNA sequencing and then transformed into *M. smegmatis* mc^2^155 as described [33]. Electrocompetent *M. marinum* and *M. ulcerans* were prepared as described [34] and these cells were transformed with 10 µg of DNA from plasmid pJKD2893 (Table S1). The constructs were confirmed to be correct in mycobacteria by Southern hybridization and back transformation to *E. coli*. Acetone soluble lipids were extracted from recombinant *M. ulcerans* and analysed by LC-MS for the presence of mycolactones as previously described [35].

### Measurement of green fluorescence protein (GFP) expression

GFP expression in pSM20 and derivatives (Table S1) was measured using a FLUOstar OPTIMA plate scanner (BMG Lab Technologies). *M. smegmatis* strains were grown to an OD of 1.0 using a WPA CO8000 cell density meter (Isogen Life Science). For each strain, 30 µl of starter culture was added to each of 16 wells of a 96-well flat-bottomed clear plate containing 150 µl of fresh 7H9 medium. Plates were incubated at 37°C for 30 mins. Each well was scanned using an excitation filter of 485 nm and an emission filter of 520 nm. Fluorescence readings were taken every 10 minutes and the average of 20 flashes per well was taken to be the measure of fluorescence. Prior to each reading, the plates were shaken for 5 minutes in an orbital motion. Replicates were averaged for each experiment and the average value for the vector-only control was taken as background and subtracted from the average at each time point.

### Preparation of total RNA

Total RNA was prepared from *E. coli* using the RNeasy mini kit as described and per the manufacturer's instructions (Qiagen) [36]. For *M. ulcerans*, a 0.5 volume of RNAlater (Qiagen) was added to 100 ml of late log-phase culture and allowed to stand at room temperature for 10 minutes prior to centrifugation at 4,600 *g*, for 10 minutes. The resultant cell pellet was washed in 1 ml of 0.5% (v/v) Tween-80 per 50 mg of cells (wet weight), resuspended in 800 µl of RNA lysis buffer (0.12 M sodium acetate (pH 4.0), 9.6% (v/v) liquid Pyroneg (Diversey), pH 4.0) and then added to 250 µg of glass beads (Sigma Aldrich), with 600 µl of acidified phenol:chloroform (pH 4.0) (Sigma Aldrich). Cells were disrupted with a FastPrep tissue homogenizer (Savant Instruments) for 45 seconds, at speed 6 and chilled on ice for 5 minutes. The aqueous phase was then re-extracted with chloroform:isoamylalcohol (24:1) and precipitated with isopropanol, and 3 M sodium acetate (pH 4.6). Two 70% (v/v) ethanol washes were performed and the pellet was dried briefly under vacuum and resuspended in 100 µl of DEPC water. RNA in this preparation was then further purified using an RNeasy extraction kit, including an on-column DNase treatment, following the manufacturers recommendations (Qiagen). For RNA extraction from *M. smegmatis* and *M. marinum* the following modifications to the above method were used. The cell pellet was first resuspended in 2 ml of lysis solution (20 mM potassium acetate (pH 4.8), 1 mM EDTA, 0.5% (v/v) SDS, 100 µg proteinase K ml^−1^). One millilitre was added to 250 µg of glass beads with 700 µl acidified phenol:chloroform pH 4.0. Cells were disrupted by three cycles in a FastPrep instrument at speed 5, for 30 seconds, and then centrifuged at 17,900 *g* for 10 minutes. The aqueous phase was recovered and extracted once with 500 µl phenol:chloroform (pH 4.0) followed by a chloroform only extraction. Nucleic acids were precipitated as above and RNA extraction proceeded as for *M. ulcerans* using the RNeasy extraction kit.

### Primer extension

The primer extension protocol used was modified from Lloyd *et al.*, [37]. Two reverse transcription reactions were performed. To the RNA-primer mix, 6 µl of 5x first strand buffer (Invitrogen), 15 mM DTT (Invitrogen), 1 mM dNTPs (Promega), 1 U RNasin (Promega) and 100 U of Superscript II RNase H^-^ reverse transcriptase (Invitrogen) were added. After one hour at 42°C, 2 µl of 5x first strand buffer, 1.5 mM dNTPs, 1 U RNasin, 15 mM DTT and 100 U of Superscript II was added and incubated for a further hour at 42°C. Ten nanograms of RNaseA (Sigma) was then added and allowed to incubate at 37°C for 30 minutes. The resultant cDNA was precipitated and washed once with 70% (v/v) ethanol, dried and stored at −20°C until analysis. Capillary electrophoresis was performed on an Applied Biosystems 3730 DNA analyzer using Liz™ 500 size standards to generate a standard curve (Applied Biosystems). Genemapper® version 3.7 (Applied Biosystems) was used to analyze the sample files with automated allele calling verified by manual inspection. The sized cDNA fragments were then mapped to their respective first strand synthesis primer binding sites to identify the putative transcription start site.

### Bioinformatic analysis

From the alignment of each of the SigA, C, D, E, F, H & L promoters [38], nucleotide frequency counts were derived and used to construct a library of 110 position specific scoring matrices (PSSMs) for each sigma factor (PSSMs available upon request). This allowed the gap between the -35 and -10 signals to vary between 14 and 23 residues, and the gap between the -10 signal and the TSP to vary between 3 and 13 residues. PoSSuM software [39] was used to scan the pMUM001 genome for high scoring hits to these PSSM libraries [40], using a background model consistent with the G+C biased nucleotide distribution of pMUM001. A *p*-value significance cutoff of 0.0001 was used.

### Site-directed mutagenesis

Splice overlap extension PCR [41] was used to alter the sequence of putative promoter motifs with oligonucleotides 1075-F and 1074-R for *mlsA1*/*mlsB*, 1667-F and 1668-R for mup045, and 1669-F and 1670-R for mup053 (Table S2). Each PCR reaction consisted of 20 cycles of 94°C for 1 minute, 50°C for 1 minute and 72°C for 3 minutes then 94°C for 1 minute, 72°C for 10 minutes and held at 4°C. Two overlapping PCR products were obtained and 2 µl (∼50 ng DNA) of each were used in a subsequent reaction using the outermost primers for each product to yield a complete fragment incorporating both products. Each product was then ligated into pSM20 as described above. Mutations were confirmed by DNA sequencing.

### Mouse-tail infections

Ten, six-week-old female BALB/c mice (Charles River France, http://www.criver.com/) were injected subcutaneously into the tail with 30 µl of a suspension containing 5×10^4^ bacteria. To favour the growth of the GFP-expressing bacilli, animals received 0.1 ml of a solution containing 80 mg/ml of kanamycin (1% w/v), administered by oral gavage every day. The mice were killed and their tails were collected fifty days after inoculation.

### Ethics statement

Mice were maintained in the animal house facility of the Centre Hospitalier Universitaire, Angers, France (Animal Ethics Committee, Centre Hospitalier Universitaire, Agreement A 49 007 002), adhering to the institution's guidelines for animal husbandry.

### Detection of cultivable bacilli and histology

The tissue specimens from mice were minced with disposable scalpels in a Petri dish and ground with a Potter–Elvehjem homogeniser, size 22, (Kimble/Kontes, Vineland, NJ), in 0.15 M NaCl to obtain a tenfold dilution. The suspension was decontaminated to remove other bacteria using an equal volume of *N*-acetyl-L-cysteine sodium hydroxide (2%) [42] and inoculated on 7H10 agar supplemented with OADC (Difco), containing 25 µg/ml of kanamycin. For histological examination, tissues were fixed in 4% paraformaldehyde in phosphate buffer (pH 7.4). Decalcification of the tissue was performed for 7 days in 0.1 M of EDTA solution in PBS. Samples were frozen in isopentane cooled to −140°C in liquid nitrogen and stored at −80°C for subsequent histochemical analysis. Eight-micron thick transverse sections were cut at −30°C on a cryostat (Jung-Reichert Cryocut 1800, Cambridge Instruments, Germany) and kept at −80°C until histochemical processing, which was done within 1 week of sectioning. For detection of GFP-expressing bacilli, tissues were counterstained with DAPI, with endogenous phosphatase activity first detected using alkaline phosphatase substrate kit I (Vector Laboratories). The preparation was then mounted in Vectashield mounting medium containing DAPI (Vector Laboratories) and the samples were visualized using fluorescence microscopy (Leica DM5000B). Hematoxylin phloxine saffron and Ziehl Nielsen staining were performed according to standard procedures.

### Mosquito microcosm *M. ulcerans-*GFP feeding experiments

Mosquito larvae (*Aedes camptorhynchus*) between first and second instar were distributed into 4×50 ml plastic tubes (10 larvae per tube), containing 20 ml of sterile tap water. To three groups of four tubes were added 1.5 ml of an aqueous slurry of possum faecal material containing either 5×10^6^ colony forming units (cfu) *M. ulcerans*-GFP, 5×10^6^ cfu *M. marinum*-GFP, or possum faecal material alone. The larvae were left to feed on the material for one week at 24°C. At the end of one week and also at the end of every subsequent week up to week five, all larvae were transferred to new tubes containing 20 ml of sterilized tap water. The original tubes spiked with possum faecal material were kept at room temperature and at the commencement of each week 500 µl of water from each of these tubes was tested by IS*2404* and *ppk* qPCR to estimate the residual quantity in the water of *M. ulcerans* and *M. marinum* respectively [43]. Results were reported as cfu by reference to standard curves for each PCR and bacterial species, correlating qPCR Ct values with cfu [43]. From weeks 2–5 the larvae were sustained with small quantities of fish food added to each tube. A larva was taken from each tube as it progressed through each instar and tested by IS*2404* PCR for the presence of *M. ulcerans* as described [43]. A selection of 4^th^ instar larvae were also fixed overnight in 10% formaldehyde in PBS (v/v) then mounted in cedarwood oil (Matheson, Coleman and Bell) on a glass slide for examination by fluorescence microscopy with an Olympus BX51 microscope (Olympus, Tokyo, Japan) with the following filter sets: DAPI (Blue) ex: 360–70 nm, em: 420–60 nm, FITC (Green) ex: 450–80 nm, em: 535 nm, TRITC (red) ex: 535 nm, em: 635 nm. Images were acquired using an Olympus DP-70 digital camera and merged using DP controller software (version 1.1.1.71) or Adobe Photoshop (version 8) These experiments were terminated before the insects progressed to pupal and adult developmental stages.

## Results

### Identification of promoter regions in mycolactone-associated genes using a GFP-reporter

**Figure 2:**
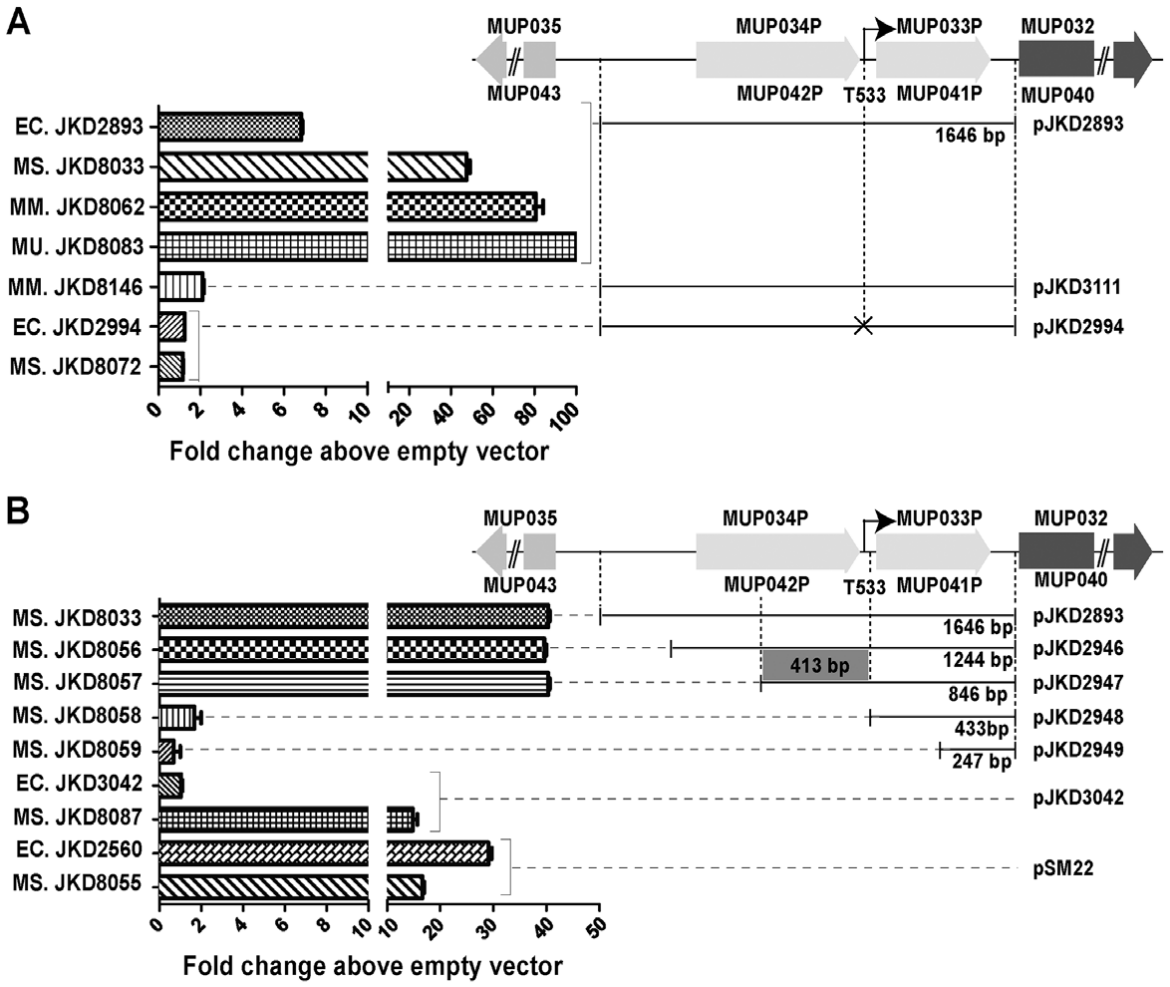
Summary of fluorescence data demonstrating the difference in promoter activity among strains containing GFP reporter constructs. Shown are plasmid constructs with upstream regions from *M. ulcerans mlsA1/mlsB* (A) & (B). Also shown are the *sigA* promoter from *M. bovis* BCG (construct pJKD3042) (B) and the *srp* promoter from *E. coli* (construct pSM22) (B). Point mutations in putative promoter regions in the putative -10 motifs of promoter regions for *mls* sequences are marked by ‘X’. All values are expressed as fold changes above the strain containing the empty vector pSM20. Strains are identifiable by a two-letter prefix to the strain number **EC** - *E. coli*, **MS** - *M. smegmatis*, **MM** - *M. marinum* and **MU** - *M. ulcerans*. ‘*’ indicates this fluorescence is more than 100-fold higher than vector alone.

By cloning DNA fragments ranging from 229 bp–1646 bp located immediately upstream of *mlsA1/mlsB* (these genes have a duplicated start and upstream sequence so one cloned fragment was sufficient to analyse both genes), *mlsA2*, mup038, mup045 and mup053 in the promoterless GFP *E. coli/Mycobacterium* reporter vector pSM20 (Table S1, Figure 1) we were able to discover regions containing promoter activities. The resulting plasmids were used to transform *E. coli*, *M. smegmatis* and, for the *mlsA1/mlsB* construct, *M. marinum* and *M. ulcerans* were also transformed. Bacteria were cultured in 96-well plates for 2 hours at 37°C and expression of GFP for each strain was assessed by continuous fluorescence measurements. *E. coli* expressing GFP from the strong, constitutive promoter *srp* (pSM22) [32] and *M. smegmatis* expressing GFP from the *sigA* promoter from *Mycobacterium bovis* BCG (pJKD3042) [44] were used as positive controls for each genus. Results were expressed as fold changes in fluorescence above the levels detected in bacteria containing the empty vector pSM20. The results for *mlsA1* and *mlsB* are summarized in Figure 2 and show that strains containing the construct pJKD2893 with the region 1646 bp upstream of *mlsA1*/*mlsB*, led to detectable GFP expression in *E. coli*, and high levels of GFP expression in *M. smegmatis* and *M. marinum* (Figure 2A). A single copy version of pJKD2893 was also created where a DNA fragment spanning the 1646 bp *mls* upstream region and *gfp* gene from pJKD2893 was subcloned into the mycobacterial integrating shuttle vector, pJKD8003 resulting in pJKD3111. *M. marinum* transformed with pJKD3111 expressed GFP 40-fold less than the same strain containing pJKD2893 (Figure 2A).

To further localize the region conferring promoter activity within the 1646 bp upstream of *mlsA1/mlsB*, four overlapping sub-clones of this region were prepared by PCR, cloned into pSM20 and used to transform *E. coli* and *M. smegmatis*. Comparison of GFP expression in these constructs in another time course experiment, comparing fluorescence with the full-length 1646 bp fragment and controls, clearly showed that promoter activity was restricted to a 413 bp fragment located between nucleotide positions 35996-36409 for *mlsA1* and 100821-101234 for *mlsB* in pMUM001 (Figure 2B).

The region 1440 bp upstream of mup045 and 1466 bp upstream of mup053 also led to significant GFP expression in *M. smegmatis*, 8–15 fold above background, but these regions showed little transcriptional activity in *E. coli* (Figure S1). No fluorescence was observed in either *E. coli* or *M. smegmatis* for strains containing the 229 bp region upstream of mup038 (pJKD3269) or the 1096 bp region upstream of *mlsA2* (pJKD3041) (data not shown). These experiments demonstrate that the regions upstream from *mlsA1/mlsB*, mup045 and mup053 all harbour at least one strong promoter.

### Identification of transcriptional start points (TSPs) for *mlsA1/mlsB*, mup045, and mup053

To identify TSPs upstream of each gene primer extension (PE) analysis was performed using RNA extracted from *M. ulcerans* Agy99. For *mlsA1/mlsB*, RNA was also extracted from *M. marinum* harbouring the GFP expression construct pJKD2893. One or more 5′ 6-FAM-labeled antisense oligonucleotides were used to prime cDNA synthesis to determine the TSP for *mlsA1/mlsB*, mup045, and mup053 (Table S2). Single, distinct PE products were identified for all three regions using multiple RNA preparations (Figure S2, Figure S3). Size fragment analysis of the PE products suggested single TSPs at 533 bp (T533) upstream of the *mlsA1*/*mlsB* translational start (Figure S2), 207 bp upstream of mup045 (T207), and 68 bp upstream of mup053 (T068) (Figure S3). Primer extension analysis of mup038 and *mlsA2* was not attempted due to the lack of promoter activity observed with the wild type sequences in the GFP reporter assays.

### Localization of putative promoter sequences by *in silico* analysis

Several studies of promoters in mycobacteria facilitated the construction of position-specific scoring matrices (PSSMs) to perform *in silico* searches for potential regulatory regions in DNA sequences [38]. We used sigma factor-specific libraries of PSSMs to scan the regions upstream of the three TSPs identified by our PE analysis. High-probability SigA-like promoter motifs were predicted in the regions upstream of *mlsA1/mlsB* and mup045 and a SigD-like motif was predicted upstream of mup053 (Table 1).

**Table 1:** Comparison of mycolactone promoter sequences with mycobacterial consensus sequences.

			* Sequence	
Promoter	Species	Gene	−35	Spacer	−10	Spacer	+1	Reference/comments
SigA	Mycobacteria	Consensus	TTGACW	17	TATAMT	6	G or A	[38],[40]
	*M. ulcerans*	*mlsA1/mlsB*	TTGCAA	19	TAAATT	13	T	Predicted by PoSSuM: *P-*value 1.875074e-04
	*M. ulcerans*	*mlsA1/mlsB*	TTGCAA		CCCATT^†^ [Table-fn nt102]			Mutagenesis of -10 box reduces transcription
	*M. ulcerans*	mup045	TCGACT	16	TACAGC	3	G	Predicted by PoSSuM: *P-*value 4.993513e-04
	*M. ulcerans*	mup045	TCGACT		GGGCCC^†^ [Table-fn nt102]			Mutagenesis of -10 box reduces transcription
SigD	Mycobacteria	Consensus	GTANCGSS	16–20	NNANNN	1	G	[40]
	*M. ulcerans*	mup053	GTATCGAC	17	ACAGGC	6	G	Predicted by PoSSuM: *P-*value 8.445395e-05
	*M. ulcerans*	mup053	GTATCGAC		GCCCGC^†^ [Table-fn nt102]			Mutagenesis of -10 box has no effect

*: R denotes A or G, S denotes C or G, M denotes A or C, W denotes A or T as per IUB ambiguity codes, N denotes any A, T, G or C.

**†:** Changed nucleotides shown in underline.

### Confirmation of putative promoter elements by site-directed mutagenesis

To confirm the *in silico* promoter predictions, the GFP expression constructs spanning the putative -10 sequences from *mlsA1/mlsB* (pJKD2893), mup045 (pJKD3040) and mup053 (pJKD3039) were mutated by PCR (Table 1). GFP production by *E. coli* and *M. smegmatis* harbouring these constructs was assayed as before by continuous fluorescence measurements over 2 hours at 37°C. Fluorescence production was compared with the same strains containing the wild-type putative promoter sequences. Mutation of the proposed −10 boxes for both the *mlsA1/mlsB* and mup045 reduced fluorescence in *M. smegmatis* to less than 4% of the wild-type sequences, strongly suggesting these sequences are functional −10 motifs, required for proper binding of the sigma factor and RNA polymerase to initiate transcription (Figure 2B). Mutation of the proposed −10 box from mup053 had no impact on GFP expression.

### Construction and analysis of *M. ulcerans* expressing GFP under the control of the *mlsA1/mlsB* promoter

The strength of the *mls* promoter led us to develop a GFP-producing strain of *M. ulcerans* that might be useful in studies of pathogenesis or transmission. We transformed *M. ulcerans* JKD8049 with plasmid pJKD2893, resulting in highly green fluorescent *M. ulcerans* (JKD8083 or *M. ulcerans*-GFP), with fluorescence expression more than 100 fold above empty vector (Figure 2A). To ensure that GFP expression did not stop mycolactone production we performed cell LC-MS analysis of acetone-soluble lipids from cultures of JKD8083 and confirmed the presence of mycolactone A/B and C (Figure S4).

### *M. ulcerans*-GFP retains virulence *in vivo*

**Figure 3:**
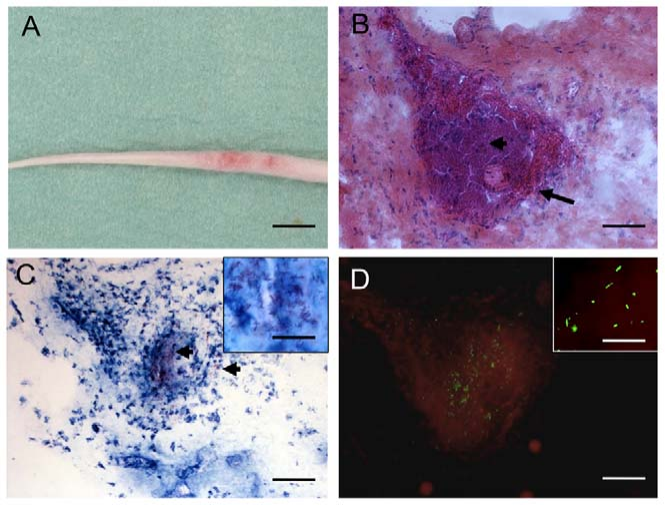
Histological study of serial mouse tail sections following mouse-tail infection with *M. ulcerans-*GFP. (A) Fifty days after subcutaneous inoculation in the tail of 10^5^ bacilli showing the beginning of ulceration. (B) Hematoxylin phloxine saffron staining on tissue section reveals massive necrosis. (C) Ziehl-Nieelsen staining showing many extracellular individual and clustered bacilli (arrowheads) associated with necrosis, and (D) bacilli expressing GFP. Scale bars: 0.8 cm (A), 100 µm (B, C, and D) and 40 µm (C and D insets).

Mouse-tail infection is a well-established animal model for studying *M. ulcerans*. Forty days after subcutaneous inoculation of 10^5^ *M. ulcerans*-GFP oedema was observed and on the 50^th^ day the lesion became ulcerated and the mice were killed. Histological study of the ulcerated region showed an area of necrosis consistent with wild type *M. ulcerans* infection (Figure 3A, Figure 3B). Granulomatous inflammation was not observed. Acid-fast bacilli were localized in clumps in necrotic areas (Figure 3C) and expressed green fluorescent protein (Figure 3D). The viability of these bacteria was demonstrated by re-isolating them in bacterial culture media. These results demonstrate that *M. ulcerans*-GFP is virulent in the mouse model and provokes lesions typical of *M. ulcerans* infection.

### *M. ulcerans*-GFP accumulates within mosquito larvae

**Figure 4:**
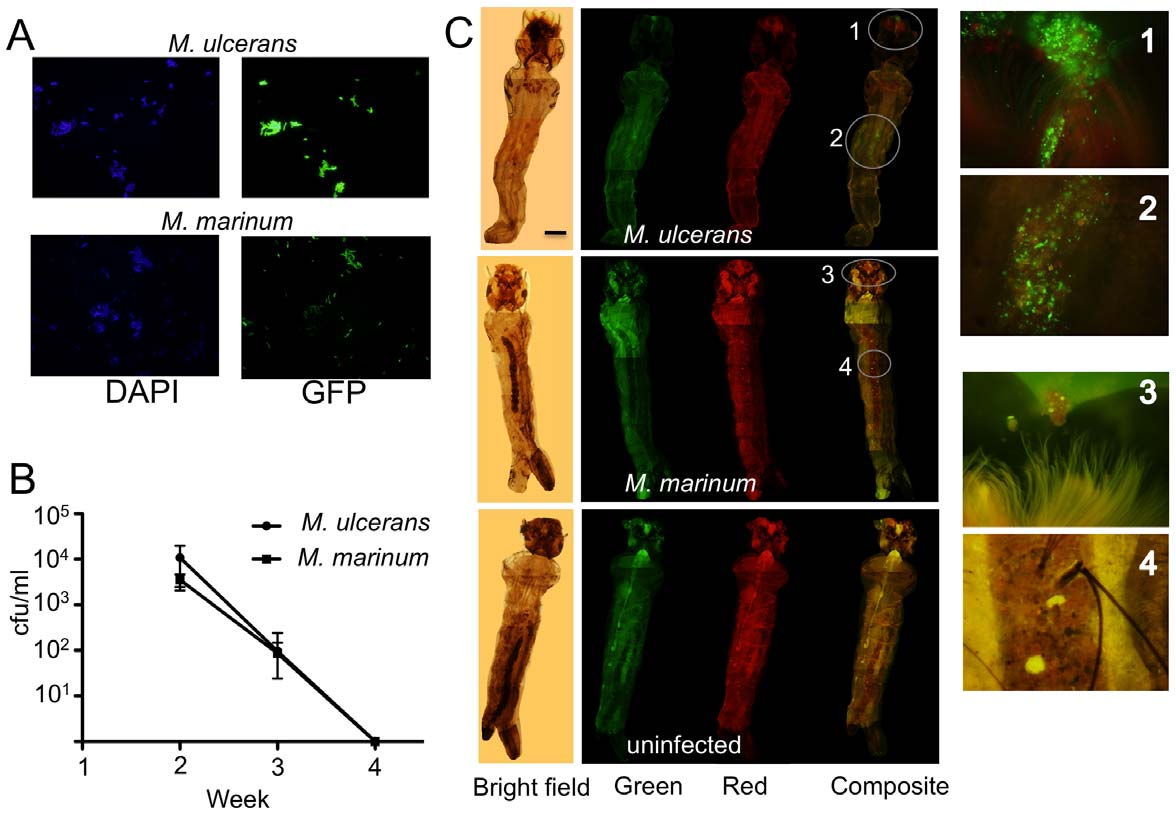
Concentration of *M. ulcerans*-GFP within 4^th^ instar mosquito larvae. (A) Epifluorescence microscopy (x100 objective) demonstrating that the majority of total fluorescent bacteria (DAPI-staining, blue filter) are also GFP-expressing *M. ulcerans* (JKD8083) and *M. marinum* (JKD8062) (green filter). (B) Reduction of *M. marinum* and *M. ulcerans* within the water of the microcosms over the 4-week duration of the experiment as measured by qPCR. Results are the means and standard deviations of four biological repeats. (C) Bright field, red fluorescence, green fluorescence and composite images showing the accumulation of *M. ulcerans* around the mouth and mid-gut of a 4^th^ instar *Aedes camptorhynchus* larva. Scale bar indicates 0.5 mm. Inset images are ×400 magnification. No specific green fluorescence was observed in larvae infected with *M. marinum* or uninfected controls. Red fluorescence images were included to show that green fluorescence was due to GFP expression and not autofluorescence.

Adult mosquitoes in some Buruli ulcer endemic regions of Australia have tested PCR positive for *M. ulcerans* and epidemiological evidence suggests a role for biting insects in the disease ecology of *M. ulcerans* [45],[46]. These data and the presence of *M. ulcerans* in possum faecal material from the same endemic regions has led to the hypothesis that larval stages of mosquitoes may and probably do ingest *M. ulcerans* as well as other bacteria via filter feeding activity on decomposing, faecally contaminated environments [47]. We mimicked this environment by establishing simple aquatic microcosms, seeded with 1 or 2 instar *Aedes camptorhynchus* larvae that were then transiently fed with possum faecal material, spiked with either *M. ulcerans*-GFP or *M. marinum*-GFP (Figure 4A). *M. ulcerans* and *M. marinum* were initially liberated into the water from the food source but neither bacterial species were detectable in water by week 4 (Figure 4B). Analysis of 4^th^ instar larvae at week 4 by fluorescence microscopy revealed an accumulation of *M. ulcerans* primarily within the larval midgut and around the mouthpart (Figure 4C). Fourth instar larvae assayed by PCR for *M. ulcerans* had a mean bacterial load of 27,300±15,200 cfu (n = 4). The same pattern of accumulation within the insect was not seen with *M. marinum*-GFP with very few fluorescent bacteria observed in association with larvae (Figure 4C). Neither *M. ulcerans* or *M. marinum* were detected in the microcosms containing mosquito larvae only (Figure 4C). These data show that mosquito larvae in contaminated aquatic environments were able to ingest and maintain *M. ulcerans* within regions of the digestive tract over a significant time period.

## Discussion

In this study we have explored gene expression of six pMUM001 genes required or implicated in mycolactone synthesis and attempted to identify their transcriptional start sites and promoter motifs.

Using a combination of primer extension and *in silico* analysis together with a GFP reporter system, we were able to identify a SigA-like promoter that drives expression of the mycolactone polyketide megasynthases *mlsA* and *mlsB* in *M. ulcerans*. Primer extension analyses with mRNA extracted from *E. coli, M. smegmatis* and *M. marinum* bearing the GFP reporter construct pJKD2893 and from wild-type *M. ulcerans* Agy99 all consistently demonstrated a transcription start point (TSP) 533 bp upstream of the *mlsA1/mlsB* initiation codons. The primer extension analysis was fully supported by the GFP expression data, wherein only strains containing expression constructs that spanned the TSP at T533 produced fluorescence. These results indicate the presence of a strong promoter preceding position T533. Sequence scanning using PoSSuM of the region immediately upstream of T533 for mycobacterial consensus promoter sequences predicted a high probability SigA-like promoter (Table 1). Site-directed mutagenesis of the putative −10 box by substitution of three nucleotides completely abolished GFP expression (Table 1, Figure 2B), implicating this sequence in RNA polymerase (RNAP) binding. The *mlsA/mlsB* promoter lies between two pseudogenes that once encoded transposases. These CDS appear to be remnants of two distinct insertion sequence elements (ISE) as the partial transposase sequences display similarity to two different IS families (IS3 family for MUP034/MUP042 and the IS6 family for MUP033/MUP041) [25]. These vestigial ISE are quite distinct to the two high copy number elements, IS*2404* and IS*2606* present in *M. ulcerans*. It is possible that the T533 promoter was once a component of an ISE. A role for ISE in altering gene expression in mycobacteria has been reported [48]. Similarly, we investigated DNA sequences upstream of mup045 and found a TSP at T207 with a potential SigA promoter element predicted by PoSSuM and confirmed by a loss of GFP expression in *M. smegmatis* following mutagenesis of the proposed −10 box. The principal mycobacterial sigma factor *sigA* is utilized by genes expressed during exponential growth [49], thus the data from *mlsA/B* and mup045 fit well with our previous report that show these genes are constantly expressed during exponential growth in the heterologous host, *M. marinum* [35].

PoSSuM sequence scanning predicted a SigA-like promoter upstream of mup045, a finding confirmed by mutagenesis of its putative −10 motif (Table 1). The same *in silico* search suggested a SigD-like promoter element upstream of mup053. However, mutation of the putative −10 motif for this gene resulted in no significant difference in GFP production in either *E. coli* and *M. smegmatis* backgrounds compared to wild type sequence, indicating that this was not the promoter region or that the introduced mutations were not sufficiently different to the wild type sequence to alter transcription. The latter scenario seems more likely given the low complexity of SigD −10 consensus sequences (Table 1).

The discovery in this study of the strong SigA-like promoter, active in diverse bacterial genera, and driving expression of the mycolactone *mls* PKS genes prompted us to transform *M. ulcerans* with a reporter plasmid with GFP under the control of the T533 *mls* promoter, resulting in the highly green fluorescent strain *M. ulcerans* JKD8049. *M. ulcerans*-GFP still produced mycolactone and was capable of causing disease in a mouse-tail infection model. Interestingly, GFP expression was more than 2-fold higher in *M. ulcerans* than in *M. marinum* harbouring the same plasmid (Figure 2A), suggesting additional regulatory factors might augment *mls* expression in *M. ulcerans* (or conversely, repress gene expression from the same promoter in other mycobacteria). The high level of *mls* promoter activity and the presence of viable *M. ulcerans*-GFP in the ulcerated tail tissue 50 days post inoculation implies that there was sustained expression of the mycolactone PKS and presumably sustained mycolactone production by the bacteria within necrotic tissue (Figure 3). These observations demonstrate the utility of this *M. ulcerans*-GFP strain as a tool for following the dynamics of *mls* gene expression during infection and understanding the role of mycolactone in pathogenesis.

We also used *M. ulcerans*-GFP to explore the previously reported association of *M. ulcerans* with *Aedes camptorhynhcus* mosquitoes [45]. Here, we addressed the specific question of whether or not *A. camptorhynchus* larvae could ingest *M. ulcerans* via feeding on possum faecal material and whether the bacteria could persist through the larval growth stages. Many larval mosquito species filter feed on microbial particles and detritus where they aggregate at air-water interfaces near plant stems and algal mats in lentic waters [50],[51] and a recent report has also suggested that *M. ulcerans* can persist within the gut of *Ochlerotatus triseriatus* mosquito larvae [52]. We were also able to observe the presence of *M. ulcerans* within the gut contents of mosquito larvae in laboratory experiments. However, the mode of larval ingestion via possum faecal pellets that we have employed in this study, presents a natural and viable pathway that *A. camptorhynchus* larvae as well as other filter-feeding macroinvertebrates might become infected for a long period of time with *M. ulcerans*. The peritrophic matrix is a proteoglycan ‘sleeve’ that separates food sources from the gut epithelium in insects [53] and our data suggests an accumulation of *M. ulcerans* within this matrix through each instar (Figure 4C). The significantly greater mean bacterial load of *M. ulcerans*-GFP found in fourth instar larvae compared to *M. marinum*-GFP may indicate that *A. camptorhynchus* larvae are able to digest and assimilate *M. marinum*-GFP better than *M. ulcerans* or that *M. ulcerans* is able to persist and perhaps multiply within the peritrophic matrix. Production of GFP in the mosquito larvae also indicates that the mycolactone *mls* genes are likely to be expressed and producing mycolactone under these conditions. Whether or not *M. ulcerans* can be transferred through larval, pupal and then adult insects remains to be tested. Experiments are now underway to examine vertical transmission of *M. ulcerans* within mosquitoes.

The data presented in this study provide the first insights into gene expression within the mycolactone biosynthesis locus and the development of *M. ulcerans*-GFP, a strain where fluorescence and toxin gene expression are linked thus providing a tool for studying Buruli ulcer pathogenesis and potential transmission to humans.

## Supporting Information

Table S1 Bacterial strains and plasmids used in this study.
(0.09 MB DOC)

Table S2 Oligonucleotides used in this study.
(0.07 MB DOC)

Figure S1 Summary of fluorescence data demonstrating the difference in promoter activity among strains containing GFP reporter plasmid constructs. Shown are upstream regions from *M. ulcerans* mup045 (A) and mup053 (B). Point mutations in putative promoter region in the putative -10 motif of the mup045 promoter region is marked by ‘X’. All values are expressed as fold changes above the strain containing the empty vector pSM20. Strains are identifiable by a two-letter prefix to the strain number EC - *E. coli*, MS - *M. smegmatis*, MM - *M. marinum* and MU - *M. ulcerans*.
(0.19 MB DOC)

Figure S2 Overview of the mls upstream region and primer extension analysis. (A) Annotation of the sequence upstream of *mlsA1/mlsB* showing the start position of the mls genes and the putative *mls* TSP (indicated by “*”) with predicted -10 and -35 promoter motifs assigned for each TSP (boxed). Representative results are shown for fluorescent PE analysis performed on RNA from (B) *M. marinum* 8062 and (C) *M. ulcerans* for *mls* using primer PE-1. Primers PE-2 and PE-LM were used to cover the entire *mls* upstream region (refer arrows in figure for primer binding positions). PE analysis curves indicate the size (sz), height (ht) and area under the curve (ar) of peaks consistently detected by DNA fragment analysis.
(0.15 MB DOC)

Figure S3 Overview of the mup045 and mup053 upstream regions and primer extension analysis. (A) Annotation of the sequence upstream of mup045 indicating the putative TSP identified (indicated by “*”) as well as putative -10 and -35 promoter sequences assigned by PoSSuM (boxed). The fluorescently labelled primer is also indicated (arrow) (B) PE analysis graphs indicate the size (sz), height (ht) and area under the curve (ar) of peaks consistently detected by DNA fragment analysis. (C) Annotation of the sequence upstream of mup053 indicating the putative TSP identified (indicated by “*”) as well as putative -10 and -35 promoter sequences assigned by PoSSuM (boxed). The fluorescently labelled primer is also indicated (arrow) (D) PE analysis graphs indicate the size (sz), height (ht) and area under the curve (ar) of peaks consistently detected by DNA fragment analysis.
(0.15 MB DOC)

Figure S4 Liquid chromatography-mass spectrometry analysis of acetone soluble lipid extracts from *M. ulcerans*-GFP. Indicated is the presence of mycolactone A/B ([M+Na]+ at *m/z* 765.5) and the presence of the nonhydroxylated mycolactone ([M+Na]+ at *m/z* 749.5). (A) Ion trace for *m/z* 765.5; (B) ion trace for m/z 749.5.
(0.10 MB DOC)
