# Correction: Mycolactone Gene Expression Is Controlled by Strong SigA-Like Promoters with Utility in Studies of *Mycobacterium ulcerans* and Buruli Ulcer

**DOI:** 10.1371/annotation/1e664e6f-3fc8-4586-a84e-73c4ec4f783f

**Published:** 2010-02-23

**Authors:** Nicholas J. Tobias, Torsten Seemann, Sacha J. Pidot, Jessica L. Porter, Laurent Marsollier, Estelle Marion, Franck Letournel, Tasnim Zakir, Joseph Azuolas, John R. Wallace, Hui Hong, John K. Davies, Benjamin P. Howden, Paul D. R. Johnson, Grant A. Jenkin, Timothy P. Stinear

Affiliations 2 and 3 were switched. Affiliation 2 should be Department of Microbiology and Immunology, University of Melbourne, Parkville, Victoria, Australia. Affiliation 3 should be Victorian Bioinformatics Consortium, Monash University, Clayton, Victoria, Australia.

