# Supplementary material for: Mycolactone Gene Expression Is Controlled by Strong SigA-Like Promoters with Utility in Studies of Mycobacterium ulcerans and Buruli Ulcer
Source: PLoS Negl Trop Dis. 2009 Nov 24;3(11):e553. doi: 10.1371/journal.pntd.0000553 (PMC2775157; doi:10.1371/journal.pntd.0000553)
Supplement: Table S1 — Bacterial strains and plasmids used in this study. (0.09 MB DOC) [file pntd.0000553.s001.doc]

**Table S1:** Bacterial strains and plasmids used in this study.

| **Species** | **Strain** | **Genotype/Comments** | **Reference** |
| --- | --- | --- | --- |
| *E. coli* | DH10B | F- *mcrA*∆ (*mrr*-*hsd*RMS-*mcr*BC) 80d*lacZ*∆M15 ∆*lacX*74 *deoR* *recA1* *araD*139 ∆(*ara, leu*)7697 *galU* *galK rpsL endA1 nupG* | Invitrogen |
|  | JKD2559 | Promoterless GFP broad host range vector | [32] |
|  | JKD2560 | Positive control, pSM22 (pSM20 with the *E. coli* *srp* promoter) | [32] |
|  | JKD2893 | DH10B containing pJKD2893 with 1646 bp of sequence upstream of *mlsA1/mlsB* | This study |
|  | JKD2946 | DH10B containing pJKD2946 with 1245 bp of sequence upstream of *mlsA1/mlsB* | This study |
|  | JKD2947 | DH10B containing pJKD2947 with 847 bp of sequence upstream of *mlsA1/mlsB* | This study |
|  | JKD2948 | DH10B containing pJKD2948 with 433 bp of sequence upstream of *mlsA1/mlsB* | This study |
|  | JKD2949 | DH10B containing pJKD2949 with 247 bp of sequence upstream of *mlsA1/mlsB* | This study |
|  | JKD2994 | DH10B containing pJKD2994 with 1646 bp of sequence upstream of *mlsA1/mlsB* and a mutated -10 region | This study |
|  | JKD3041 | DH10B containing pJKD3041 with 1096 bp sequence upstream of *mlsA2* | This study |
|  | JKD3042 | pSM20 with the *M. bovis* BCG *sigA* promoter | This study |
|  | JKD3040 | DH10B containing pJKD3040 with 1440 bp of sequence upstream of mup045 | This study |
|  | JKD3204 | DH10B containing pJKD3204 with 1440 bp of sequence upstream of mup045 and a mutated -10 region | This study |
|  | JKD3039 | DH10B containing pJKD3039 with 1466 bp of sequence upstream of mup053 | This study |
|  | JKD3205 | DH10B containing pJKD3205 with 1466 bp of sequence upstream of mup053 and a mutated -35 region | This study |
|  | JKD3269 | DH10B containing pJKD3269 with 229 bp upstream of mup038 | This study |
|  | JKD8003 | Mycobacterial integrating vector | [25] |
|  | JKD3111 | DH10B containing *mls* promoter and *gfp* from pJKD2893 cloned into *NsiI* site of pJKD8003 | This study |
| *M. smegmatis* | mc^2^155 |  | [33] |
|  | JKD8032 | mc^2^155 containing promoterless GFP broad host range vector | This study |
|  | JKD8055 | mc^2^155 containing pSM20 with the *E. coli* *srp* promoter | This study |
|  | JKD8033 | mc^2^155 containing pJKD2893 with 1646 bp of sequence upstream of *mlsA1/mlsB* | This study |
|  | JKD8056 | mc^2^155 containing pJKD2946 with 1245 bp of sequence upstream of *mlsA1/mlsB* | This study |
|  | JKD8057 | mc^2^155 containing pJKD2947 with 847 bp of sequence upstream of *mlsA1/mlsB* | This study |
|  | JKD8058 | mc^2^155 containing pJKD2948 with 433 bp of sequence upstream of *mlsA1/mlsB* | This study |
|  | JKD8059 | mc^2^155 containing pJKD2949 with 247 bp of sequence upstream of *mlsA1/mlsB* | This study |
|  | JKD8072 | mc^2^155 containing pJKD2994 with 1646 bp of sequence upstream of *mlsA1/mlsB* and a mutated -10 region | This study |
|  | JKD8087 | Positive control, pSM20 with the *M. bovis* BCG *sigA* promoter | This study |
|  | JKD8085 | mc^2^155 containing pJKD3040 with 1440 bp of sequence upstream of mup045 | This study |
|  | JKD8139 | mc^2^155 containing pJKD3204 with 1440 bp of sequence upstream of mup045 and a mutated -10 region | This study |
|  | JKD8084 | mc^2^155 containing pJKD3039 with 1466 bp of sequence upstream of mup053 | This study |
|  | JKD8140 | mc^2^155 containing pJKD3205 with 1466 bp of sequence upstream of mup053 and a mutated -35 region | This study |
| *M. marinum* | KSW1 | Spontaneous non-pigmented mutant | L. Ramakrishnan |
|  | JKD8062 | KSW1 containing pJKD2893 with 1646 bp of sequence upstream of *mlsA1/mlsB* | This study |
|  | JKD8146 | KSW1 containing integrated pJKD3111 with *mls* promoter and *gfp* from pJKD2893 | This study |
| *M. bovis* BCG | 06164878 | Human clinical isolate | VIDRL† |
| *M. ulcerans* | Agy99 | Human clinical isolate. Genome sequence available (Accession No. NC_008611). | [26] |
|  | 04126204 | Human clinical isolate from South East Australia | VIDRL |
|  | JKD8083 | *M. ulcerans* 04126204 containing pJKD2893 with 1646 bp of sequence upstream of *mlsA1/mlsB* | This study |

† Obtained from Victorian Infectious Diseases Reference Laboratory
