# Supplementary material for: Mycolactone Gene Expression Is Controlled by Strong SigA-Like Promoters with Utility in Studies of Mycobacterium ulcerans and Buruli Ulcer
Source: PLoS Negl Trop Dis. 2009 Nov 24;3(11):e553. doi: 10.1371/journal.pntd.0000553 (PMC2775157; doi:10.1371/journal.pntd.0000553)
Supplement: Table S2 — Oligonucleotides used in this study. (0.07 MB DOC) [file pntd.0000553.s002.doc]

**Table S2:** Oligonucleotides used in this study

| **Primer** | **Position in pMUM001**† | **Oligonucleotide (5’-3’)^*^** | **Function** |
| --- | --- | --- | --- |
| 582-F | 35197-35215 | GC**GCATGC**GGTTGTCCTTCCTGTCGTC | PCR of the 1646 bp region upstream of the *mls* genes for ligation into pSM20 resulting in pJKD2893 |
| 584-R | 36823-36842 | GC**GATATC**TAGCGCAGTTGGCTGTAGTG |  |
| 701-F | 35599-35616 | GC**GCATGC**CTAGTCCGCCAAACACCT | PCR of the 1245 bp region upstream of the *mls* genes for ligation into pSM20 resulting in pJKD2946 |
| 584-R | 36823-36842 | GC**GATATC**TAGCGCAGTTGGCTGTAGTG |  |
| 703-F | 35997-36012 | GC**GCATGC**CAAGCGCTGCGACAGT | PCR of the 847 bp region upstream of the *mls* genes for ligation into pSM20 resulting in pJKD2947 |
| 584-R | 36823-36842 | GC**GATATC**TAGCGCAGTTGGCTGTAGTG |  |
| 705-F | 36410-36427 | GC**GCATGC**AATGTTAACCGGTGGTGC | PCR of the 433 bp region upstream of the *mls* genes for ligation into pSM20 resulting in pJKD2948 |
| 584-R | 36823-36842 | GC**GATATC**TAGCGCAGTTGGCTGTAGTG |  |
| 707-F | 36597-36614 | GC**GCATGC**TGAGGTGTTGGTGAAGGT | PCR of the 247 bp region upstream of the *mls* genes for ligation into pSM20 resulting in pJKD2949 |
| 584-R | 36823-36842 | GC**GATATC**TAGCGCAGTTGGCTGTAGTG |  |
| 1113-F | 2971179-  2971198^c^ | GC**GCATGC**ATCTGGCCTGACCTGTTCAT | PCR of the region upstream of the *sigA* gene from *M. bovis* BCG for ligation into pSM20 resulting in pJKD3042 |
| 1112-R | 2971518-2971499^#^ | GC**GATATC**TACACCCCTTCGGTCGTTAC |  |
| 1147-F | 34949-34951 | AA**CTGCAG**CGTCGAGGTGCTTGAGGT | PCR of the 1440 bp region upstream of mup045 for ligation into pSM20 resulting in pJKD3040 |
| 1152-R | 33554-33574 | GC**GGATCC**CTGGTTCTCCGATATCTCGTT |  |
| 1663-F | 23829-23848 | GC**GCATGC**GGTGTCGAAGGCATCTAAGG | PCR of the 1466 bp region upstream of mup053 for ligation into pSM20 resulting in pJKD3039 |
| 1664-R | 25275-25294 | GC**GATATC**GGCCTCCTTCGGTAAATACA |  |
| 1341-F |  | CCA**ATGCAT**CAAGCGCTGCGACAGT | PCR of *mls* promoter and *gfp* from pJKD2893 to clone into pJKD8003 |
| 1342-R |  | CCA**ATGCAT**CGTTTCACTTCTGAGTTCGGC |  |
| PE-LM | 36919-36948 | CTGCGACTGCATCACCCAACACCCGACCTC | 5’ FAM labelled for use in primer extension analysis located within the load module domain of *mlsA1/mlsB* |
| PE-1 | 36499-36528 | TAGTCCTTCCAGGTCGCCGATGCACACCAA | 5’ FAM labelled for use in primer extension analysis for *mlsA1/mlsB* |
| PE-2 | 36620-36645 | CGCACGCCACAGACAACGGGTGATCC | 5’ FAM labelled for use in primer extension analysis for *mlsA1/mlsB* |
| PE-m45 | 33517-33543 | CCGTTCCACTTATGTAGATGTCATTCC | 5’ FAM labelled for use in primer extension analysis for mup045 |
| PE-m53 | 25307-25336 | GCAACCCGTGCGCCGCAATCCAGTTCAATC | 5’ FAM labelled for use in primer extension analysis for mup053 |
| 1075-F | 36356-36391 | CAGTGGGTGGAGTTCG**GGGCCC**ATTGGGCTGCGAAG | PCR with 584-R for use in splice overlap extension |
| 1074-R | 36356-36391 | CTTCGCAGCCCAAT**GGGCCC**CGAACTCCACCCACTG | PCR with 582-F for use in splice overlap extension |
| 1667-F | 33746-33781 | CCGCAACGGCGGACC**GGGCCC**ATGGGCACAGATCAG | PCR with 1152-R for use in splice overlap extension of mup045 |
| 1668-R | 33746-33781 | CTGATCTGTGCCCAT**GGGCCC**GGTCCGCCGTTGCGG | PCR with 1147-F for use in splice overlap extension of mup045 |
| 1712-F | 25180-25215 | gacaaaggtgcgatg**gggccc**gcacagacgacgccg | PCR with 1711-R for use in splice overlap extension of mup053 |
| 1711-R | 25180-25215 | cggcgtcgtctgtgc**gggccc**catcgcacctttgtc | PCR with 1712-F for use in splice overlap extension of mup053 |

†Reference relates to sequence upstream of *mlsA1* on the forward strand of pMUM001. Identical sequence can be found upstream of *mlsB.*

^*^Introduced *SphI*, *EcoRV* and *ApaI* restriction enzyme sites are indicated in bold type.

^#^refers to genome coordinates in *M. bovis* BCG Pasteur (Acc No. NC_008769)

¶ refers to genome coordinates in *M. ulcerans* Agy99 (Acc No. NC_008611)
