# Supplementary material for: Mycolactone Gene Expression Is Controlled by Strong SigA-Like Promoters with Utility in Studies of Mycobacterium ulcerans and Buruli Ulcer
Source: PLoS Negl Trop Dis. 2009 Nov 24;3(11):e553. doi: 10.1371/journal.pntd.0000553 (PMC2775157; doi:10.1371/journal.pntd.0000553)
Supplement: Figure S1 — Summary of fluorescence data demonstrating the difference in promoter activity among strains containing GFP reporter plasmid constructs. Shown are upstream regions from M. ulcerans mup045 (A) and mup053 (B). Point mutations in putative promoter region in the putative -10 motif of the mup045 promoter region is marked by ‘X’. All values are expressed as fold changes above the strain containing the empty vector pSM20. Strains are identifiable by a two-letter prefix to the strain number EC - E. coli, MS - M. smegmatis, MM - M. marinum and MU - M. ulcerans. (0.19 MB DOC) [file pntd.0000553.s003.doc]

**Figure S1**


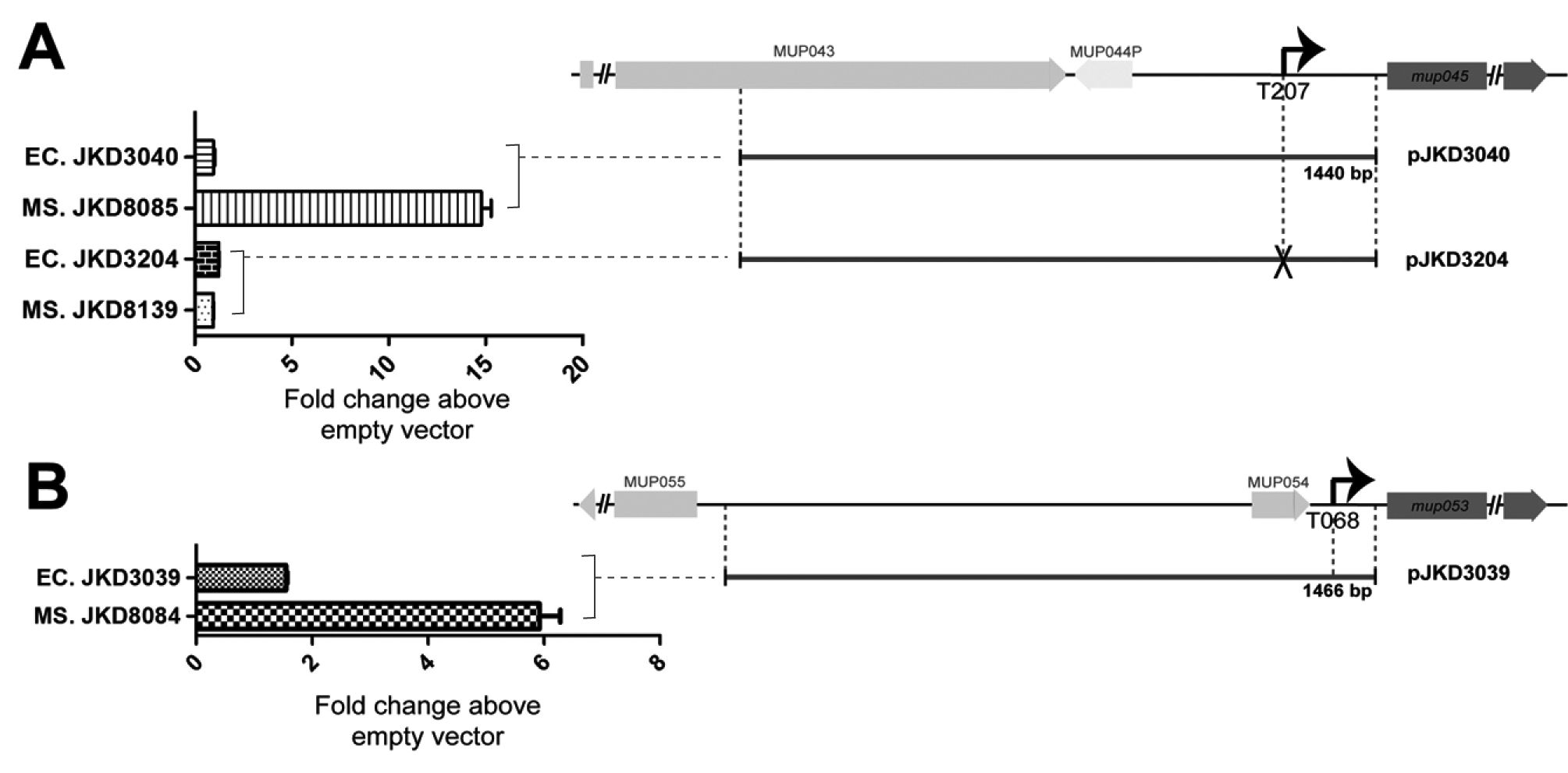


**Figure S1.** Summary of fluorescence data demonstrating the difference in promoter activity among strains containing GFP reporter plasmid constructs. Shown are upstream regions from *M. ulcerans* mup045 (A) and mup053 (B). Point mutations in putative promoter region in the putative -10 motif of the mup045 promoter region is marked by ‘X’*.* All values are expressed as fold changes above the strain containing the empty vector pSM20. Strains are identifiable by a two-letter prefix to the strain number **EC -** *E. coli*, **MS -** *M. smegmatis*, **MM** - *M. marinum* and **MU** - *M. ulcerans*.
