# Supplementary material for: Mycolactone Gene Expression Is Controlled by Strong SigA-Like Promoters with Utility in Studies of Mycobacterium ulcerans and Buruli Ulcer
Source: PLoS Negl Trop Dis. 2009 Nov 24;3(11):e553. doi: 10.1371/journal.pntd.0000553 (PMC2775157; doi:10.1371/journal.pntd.0000553)
Supplement: Figure S2 — Overview of the mls upstream region and primer extension analysis. (A) Annotation of the sequence upstream of mlsA1/mlsB showing the start position of the mls genes and the putative mls TSP (indicated by “*”) with predicted -10 and -35 promoter motifs assigned for each TSP (boxed). Representative results are shown for fluorescent PE analysis performed on RNA from (B) M. marinum 8062 and (C) M. ulcerans for mls using primer PE-1. Primers PE-2 and PE-LM were used to cover the entire mls upstream region (refer arrows in figure for primer binding positions). PE analysis curves indicate the size (sz), height (ht) and area under the curve (ar) of peaks consistently detected by DNA fragment analysis. (0.15 MB DOC) [file pntd.0000553.s004.doc]

**Figure S2**

**
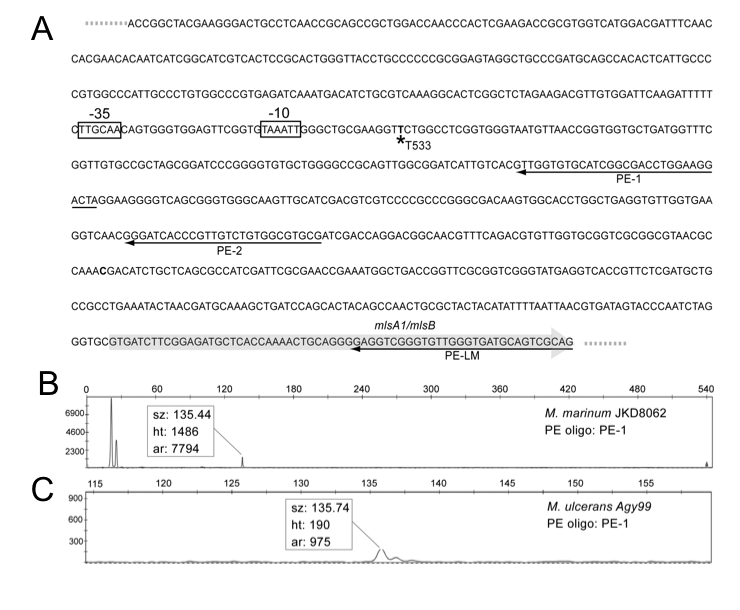
**

**Figure S2.** Overview of the *mls* upstream region and primer extension analysis. (A) Annotation of the sequence upstream of *mlsA1*/*mlsB* showing the start position of the *mls* genes and the putative *mls* TSP (indicated by “*”) with predicted -10 and -35 promoter motifs assigned for each TSP (boxed). Representative results are shown for fluorescent PE analysis performed on RNA from (B) *M. marinum* 8062 and (C)*M. ulcerans* for *mls* using primer PE-1. Primers PE-2 and PE-LM were used to cover the entire *mls* upstream region (refer arrows in figure for primer binding positions). PE analysis curves indicate the size (sz), height (ht) and area under the curve (ar) of peaks consistently detected by DNA fragment analysis.
