# Supplementary material for: Mycolactone Gene Expression Is Controlled by Strong SigA-Like Promoters with Utility in Studies of Mycobacterium ulcerans and Buruli Ulcer
Source: PLoS Negl Trop Dis. 2009 Nov 24;3(11):e553. doi: 10.1371/journal.pntd.0000553 (PMC2775157; doi:10.1371/journal.pntd.0000553)
Supplement: Figure S3 — Overview of the mup045 and mup053 upstream regions and primer extension analysis. (A) Annotation of the sequence upstream of mup045 indicating the putative TSP identified (indicated by “*”) as well as putative -10 and -35 promoter sequences assigned by PoSSuM (boxed). The fluorescently labelled primer is also indicated (arrow) (B) PE analysis graphs indicate the size (sz), height (ht) and area under the curve (ar) of peaks consistently detected by DNA fragment analysis. (C) Annotation of the sequence upstream of mup053 indicating the putative TSP identified (indicated by “*”) as well as putative -10 and -35 promoter sequences assigned by PoSSuM (boxed). The fluorescently labelled primer is also indicated (arrow) (D) PE analysis graphs indicate the size (sz), height (ht) and area under the curve (ar) of peaks consistently detected by DNA fragment analysis. (0.15 MB DOC) [file pntd.0000553.s005.doc]

**Figure S3**

**
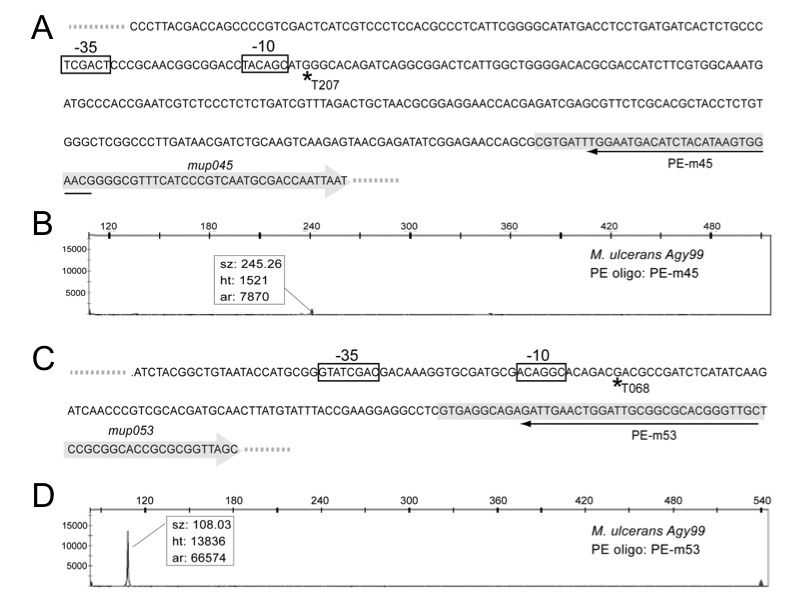
**

**Figure S3** Overview of the mup045 *and* mup053 upstream regions and primer extension analysis. (A) Annotation of the sequence upstream of mup045 indicating the putative TSP identified (indicated by “*”) as well as putative -10 and -35 promoter sequences assigned by PoSSuM (boxed). The fluorescently labelled primer is also indicated (arrow) (B) PE analysis graphs indicate the size (sz), height (ht) and area under the curve (ar) of peaks consistently detected by DNA fragment analysis. (C) Annotation of the sequence upstream of mup053 indicating the putative TSP identified (indicated by “*”) as well as putative -10 and -35 promoter sequences assigned by PoSSuM (boxed). The fluorescently labelled primer is also indicated (arrow) (D) PE analysis graphs indicate the size (sz), height (ht) and area under the curve (ar) of peaks consistently detected by DNA fragment analysis.
