# Supplementary material for: Mycolactone Gene Expression Is Controlled by Strong SigA-Like Promoters with Utility in Studies of Mycobacterium ulcerans and Buruli Ulcer
Source: PLoS Negl Trop Dis. 2009 Nov 24;3(11):e553. doi: 10.1371/journal.pntd.0000553 (PMC2775157; doi:10.1371/journal.pntd.0000553)
Supplement: Figure S4 — Liquid chromatography-mass spectrometry analysis of acetone soluble lipid extracts from M. ulcerans-GFP. Indicated is the presence of mycolactone A/B ([M+Na]+ at m/z 765.5) and the presence of the nonhydroxylated mycolactone ([M+Na]+ at m/z 749.5). (A) Ion trace for m/z 765.5; (B) ion trace for m/z 749.5. (0.10 MB DOC) [file pntd.0000553.s006.doc]

**Figure S4**

**
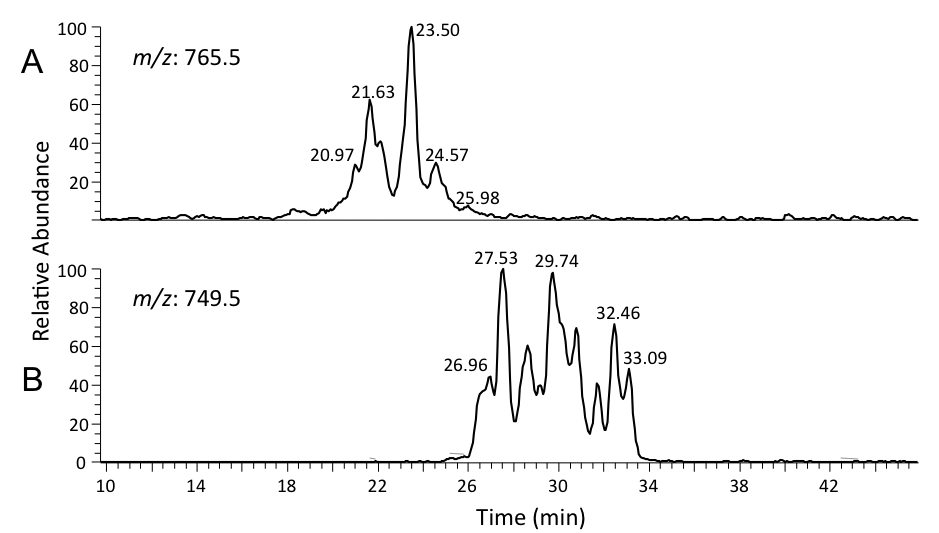
**

**Figure S4**. 4. Liquid chromatography-mass spectrometry analysis of acetone soluble lipid extracts from *M. ulcerans*-GFP. Indicated is the presence of mycolactone A/B ([M+Na]+ at *m/z* 765.5) and the presence of the nonhydroxylated mycolactone ([M+Na]+ at *m/z* 749.5). (A) Ion trace for *m/z* 765.5; (B) ion trace for *m/z* 749.5.
